# Understanding cutaneous tuberculosis: two clinical cases

**DOI:** 10.1099/jmmcr.0.005070

**Published:** 2016-12-16

**Authors:** Flavio De Maio, Enrico Maria Trecarichi, Elena Visconti, Maurizio Sanguinetti, Giovanni Delogu, Michela Sali

**Affiliations:** ^1^​ Insitute of Microbiology, Università Cattolica del Sacro Cuore, Rome, Italy; ^2^​ Institute of Infectious Diseases, Università Cattolica del Sacro Cuore, Rome, Italy

**Keywords:** cutaneous, diagnosis, infection, mycobacterium, tuberculosis

## Abstract

Tuberculosis (TB) is an ancient human disease and remains today one of the most important public health problems and the second most frequent cause of death from an infectious disease worldwide. While pulmonary TB is the most common form, extra-pulmonary TB is on the rise due to the increase in immunosuppressed subjects. Cutaneous TB manifestations are rare forms of extra-pulmonary TB due to systemic dissemination of bacilli or direct inoculation, involving skin or skin-associated tissue, more common in immunocompromised subjects. Some risk factors and the features of the lesion may prompt the suspicion of cutaneous TB, but only microbiological assays can confirm the diagnosis. Our work summarizes cutaneous TB manifestations and differences from other skin mycobacterial infections, also describes two characteristic clinical cases.

## Introduction

Tuberculosis (TB) has accompanied mankind since prehistoric time and remains today one of the most important global public health problems and the second most frequent cause of death from a single infectious agent worldwide, (World Health Organization, 2014). The World Health Organization (WHO) TB global report estimates 9 million the new cases per year (6.1 million notified to national TB control programs) and 1.5 million deaths in 2013, with the highest burden in South-East Asia, Pacific regions and Sub-Saharan Africa (World Health Organization, 2014). Although pulmonary TB is the most frequent form, estimates indicate that there are 0.8 million extra-pulmonary TB cases that are bacteriologically confirmed or clinically diagnosed (22 % of European notified TB cases) (Solovic *et al.*, 2013; World Health Organization, 2014). TB lymphadenitis is the most common extra-pulmonary form, but TB occurs also in the pleura, urogenital tract, bones and joints, central nervous system, bowel, peritoneum, pericardium and skin (Solovic *et al.*, 2013; World Health Organization, 2014). Cutaneous TB is a rare form (1–2 % of all TB cases) in Western countries but remains a significant problem in high-prevalence countries (Bravo & Gotuzzo, 2007).

Cutaneous TB may be considered an atypical, rare and heterogeneous skin infection caused by members of the *Mycobacterium tuberculosis* complex, a group of mycobacteria that cause TB in mammals (Dias *et al.*, 2014; Lai-Cheong *et al.*, 2007). Cutaneous TB mainly affects immunocompromised individuals, as highlighted by the high incidence in human immunodeficiency virus (HIV)-infected subjects and in patients undergoing immunosuppressive therapies (Handog *et al.*, 2008; Santos *et al.*, 2014). Moreover, it has been observed that cutaneous TB may emerge as a complication following immune reconstitution caused by antiretroviral therapy (Huiras *et al.*, 2008; Robertson *et al.*, 2006) and an increased risk of cutaneous TB was also associated with pregnancy (Böddinghaus *et al.*, 2007; Good *et al.*, 1981).

A major challenge is the differential diagnosis of cutaneous TB from other skin infections such as leishmaniasis, leprosy, chromomycosis, sporotrichosis and granulomatous and verrucous lesions of different origins (Bhutto *et al.*, 2002), though cutaneous infections caused by non-tuberculous mycobacteria (NTM) are those that can be more often confused with cutaneous TB (Mitha *et al.*, 2011) because of the clinical features and population target. It has been noted that NTM infections may develop following traumatic injury, surgery or cosmetic procedures (Bhambri *et al.*, 2009; Hautmann & Lotti, 1994; Liao *et al.*, 2007; Lotti & Hautmann, 1993).

The incidence of cutaneous NTM infections rose in the past 30 years from 0.7 per 100 000 to 2 per 100 000 person-years, with no clear association with sex and/or age (Wentworth *et al.*, 2013). The most frequent NTM involved in cutaneous infections are *M. fortuitum*, *M. avium*, *M. gordonae*, *M. chelonae*, *M. abscessus*, *M. kansasii*, *M. leprae* and *M. ulcerans* (Aboutalebi *et al.*, 2012; Bhambri *et al.*, 2009; El-Khalawany, 2014; Liao *et al.*, 2007; Böddinghaus *et al.*, 2007; Ridley & Jopling, 1966), with lesions generally localized in the upper limbs and peripheral parts of the body, where the lower body temperature matches well with their optimal growth temperature, as classically highlighted for *M. marinum* infections (Patel *et al.*, 2014; Tebruegge & Curtis, 2011).

Cutaneous TB may emerge as an exogenous infection, when bacilli originating usually from a patient with active pulmonary TB enter the skin tissue through small lesions, similarly to the pattern that has been observed for NTM skin infections (Bravo & Gotuzzo, 2007). In this case, once *M. tuberculosis* reaches skin and soft tissues, it can resist host immune responses and start replicating and causing the classical granulomatous lesions which may evolve in cutaneous TB. In contrast, endogenous cutaneous TB is caused by reactivation of latent TB infection (LTBI) which occurs in the skin and soft tissues years or decades following primary infection (Bravo & Gotuzzo, 2007). After infection with *M. tuberculosis*, the human host usually can control bacterial replication and prevent the development of overt disease, though the bacilli, following early replication in the lung, can spread through the lymphatic system and bloodstream to all tissues, where they can persist for years or decades (Bishai, 2000; Ottenhoff & Kaufmann, 2012; Wolf *et al.*, 2008). During this time, a dynamic equilibrium between *M. tuberculosis* and the host immune response is established and bacilli are thought to be present in the lung parenchyma as well as in many other organs and tissues, with a specific tropism for the fat tissue (Barry *et al.*, 2009; Neyrolles *et al.*, 2006). It is estimated that this state of LTBI occurs in 90–95 % of the infected subjects and can last for lifetime, with bacilli continuously replicating and capable of stimulating the host immune response that can control bacterial growth but is unable to eradicate the infection (Chao & Rubin, 2010). When the host immune response fails to control bacterial replication, such as in immune-deficient subjects or for yet unknown reasons (Chao & Rubin, 2010; Gengenbacher & Kaufmann, 2012; Lowe *et al.*, 2012), active bacterial replication ensues and granulomatous lesions may start organizing in different tissues such as skin, thus eventually leading to the classical features of cutaneous TB. Since TB is a very complex disease and we lack a sufficient understanding of the molecular and immunological mechanisms of its pathogenesis, many are the factors that can contribute to defining the type and extent of the lesions and the clinical manifestations of cutaneous TB. Among them, probably the host immune status, the features and genotype of *M. tuberculosis*, the size of the inoculum and the regions of the body involved represent the most important factors.

Despite several forms of cutaneous TB having been described and classified, as shown in Table 1, the main features of the macroscopic lesions (ulcers, plaques, cutaneous rash) and the microscopic structures as observed during histopathological analysis (characterized by the presence of the tuberculoid granuloma), highlight a common theme which is not recapitulated by the clinical classification. On the contrary, the regions involved and the host’s immune status appear to be important traits for the above mentioned classification. In this context, it is worth mentioning that classification of cutaneous TB has been proposed mainly by dermatologists, who primarily focused their attention on clinically pertinent features. Since some clinical manifestations indicated in Table 1 may be caused by NTM or other infectious agents, final diagnosis of cutaneous TB requires the identification of *M. tuberculosis* in the clinical sample.

**Table 1.** Summary of different cutaneous tuberculosis manifestations

	Clinical forms	Subjects at risk	Body parts involved	Lesion features	Histopathology	TST
Exogenous	Tuberculosis chancre	Unvaccinated children, contacts with pulmonary TB patients	Face and limbs Surgical wounds Tattoos and piercing sites	Shallow Painless ulcer Painful regional lymphadenopathy Fistulae Erythema nodosum	Acute neutrophilic infiltrate with necrotic area that becomes a granuloma with giant cells	Negative but becomes positive during disease evolution
	Tuberculosis verrucosa cutis	Health workers, contacts with pulmonary TB patients	Extremities	Verrucous and tuberous papules Adenopathy	Pseudoepitheliomatous hyperplasia Hyperkeratosis Tuberculoid granuloma	Strongly positive
Endogenous	Lupus vulgaris	Previously sensitized individuals	Face Rarely mucosae	Papulonodular lesions Plaques Ulcers	Pseudoepitheliomatous hyperplasia Tuberculoid granuloma with rare caseous necrosis	Positive
	Scrufuloderma	Children and young people	Cervical and inguinal regions	Nodules Gumma Ulcers	Tuberculoid granuloma with caseous necrosis	Strongly positive
	Orificial tuberculosis	Immunocompromised patients	Mucosae of natural orifices	Painless ulcers	Tuberculoid granuloma with necrosis and ulceration	Negative
	Acute cutaneous military tuberculosis	Immunocompromised patients and anergic children	Trunk	Erithematous papulovescicular lesions Exanthematous rash	Tuberculoid granuloma with necrosis and ulceration	Negative
Tuberculids	Papulonecrotic tuberculid	Children and young people	Lower and upper limbs Buttocks	Erithematous papulonodular lesions	Leukocytoclastic vasculitis Tuberculoid granuloma	Positive
	Lichenoid tuberculid	Children	Trunk	Perifollicular erythematous papulaes	Superficial granulomas with little or no caseous necrosis	Strongly positive
	Erythema induratum of Bazin	Previously sensitized young women	Lower limbs	Erithematous nodules and plaques	Tuberculoid granuloma with caseous necrosis Vascular alterations	Positive

The hallmarks of the cutaneous lesions together with an accurate patient anamnesis, which includes assessment of *M. tuberculosis* infection status, may make the case for potential cutaneous TB. Immunological diagnosis of TB infection is classically obtained with the tuberculin skin test (TST). Patients with cutaneous TB usually show a positive TST, even if NTM infections may also lead to a positive TST. Moreover, the TST assay is characterized by a low specificity, particularly in TB-endemic countries where cutaneous TB is more often diagnosed, due to NTM sensitization and/or BCG immunization (Delogu & Goletti, 2014). In the last 15 years, interferon (IFN)-γ release assays (IGRA), that measure IFN-γ release after stimulation of peripheral T cells with specific *M. tuberculosis* antigens, have been widely used as surrogates for TST. Two IGRAs are commercially available: T-SPOT TB (Oxford Immunotec), which relies on the stimulation of PBMC and QuantiFERON TB Gold In-Tube (QFT-GIT; Qiagen), where stimulation is carried out directly on whole blood. In both assays, a T cells are stimulated with a mixture of synthetic peptides corresponding to epitopes of highly immunogenic proteins (ESAT-6, CFP-10 and TB7.7), encoded by RD regions in the *M. tuberculosis* genome (that is genomic regions that are missing in BCG). These two assays have shown increased sensitivity and specificity compared with TST and offer the opportunity to assess the host immune status by measuring T cell reactivity against a mitogen (Sester *et al.*, 2011; Delogu & Goletti, 2014). Unfortunately, both assays do not have prognostic value and cannot distinguish LTBI subjects from patients with active TB.

Histological analysis of biopsy material may be useful for distinguishing cutaneous TB from other NTM mycobacterial skin infections (Bartralot *et al.*, 2000, 2005; Min *et al.*, 2012; Ranawaka *et al.*, 2010). It is well described that in cutaneous TB, granulomas are usually observed in the upper and mid dermis, with the presence of caseous necrosis in addition to well-formed epithelioid cells containing Langhans giant cells and lymphocytes (Bhutto *et al.*, 2002). In contrast, in NTM cutaneous infections, a greater neutrophilic infiltration with interstitial granulomas and small vessel proliferation are observed (Min *et al.*, 2012).

Final diagnosis of cutaneous TB is classically achieved by the microbiological detection of *M. tuberculosis* in a biopsy specimen. The presence of acid-fast bacilli (AFB) in the specimen subjected to Ziehl–Nielsen staining cannot be used to distinguish cutaneous TB from NTM infections and, due to the paucibacillary nature of most cases of cutaneous TB, AFB cannot be readily observed in the clinical specimen. Isolation of *M. tuberculosis* in culture remains the gold standard, though it requires up to 5–6 weeks and may end up with false negative results. Detection of the *M. tuberculosis* genome could represent the most effective and rapid tool to make diagnosis, using one of the several DNA amplification techniques available (Sali *et al.*, 2015). It has been suggested that these techniques, while capable of detecting a small number of copies of the mycobacterial genome, may not have good sensitivity in these settings because of the non-uniform distribution of mycobacteria and the presence of inhibitory substances in the tissue specimens (Mehta *et al.*, 2012). Recent advancements in DNA extraction and purification from tissues are improving the performance of these assays and today many commercial systems are available, some of which offer the possibility to simultaneously detect *M. tuberculosis* and NTM, providing an opportunity for the differential diagnosis of cutaneous mycobacterial infections (Min *et al.*, 2012).

Traditionally, the treatment of cutaneous TB disease follows the same guidelines as pulmonary TB (Dartois, 2014) with two-months quadruple-regime therapy (isoniazid, rifampicin, pyrazinamide and ethambutol) followed by four-months of double therapy (isoniazid and rifampicin) (Ramam *et al.*, 2005, 2007; Wang *et al.*, 2011). Longer treatments are indicated when cutaneous TB is associated with other extra-pulmonary TB manifestations (Bravo & Gotuzzo, 2007). Generally, 4–6 weeks are necessary to obtain a good clinical response, but unsuccessful treatment may occur, with the presence of drug-resistant strains requiring the use of the less effective second-line drugs (e.g. capreomycin, kanamycin, ethionamide). Monitoring of drug toxicity and patient compliance are essential, as for pulmonary TB (Handog *et al.*, 2008).

In this study, we present two clinical cases of cutaneous TB which emphasize the complexity of the disease.

## Case reports

### Patient 1

A 26-year-old woman, originating from Bangladesh and living in Italy for about 2 years and who had given birth 4 months previously, presented with a phlegmon episode of the fourth finger of the right hand consequent to a domestic incident with an herringbone dating back to 2 months earlier. She was admitted to the Orthopedic and Hand Surgery Unit of Gemelli Hospital (Rome, Italy). Laboratory parameters were normal. At systemic examination no abnormalities were evident except for a macroscopic soft tissue tumefaction of the fourth finger of the right hand. Surgical drainage was performed followed by antibiotic treatment with ciprofloxacin and amoxicillin/clavulanic acid for a presumptive bacterial infection, which did not result in clinical improvement. Routine blood microbiological investigations and drainage liquid culture gave negative results for pathogenic bacteria. The patient reported an episode of chest pain about 4 months before the present hospitalization and recurrent episodes of non-productive cough (she did not report fever, sweating or weight loss). A chest X-ray showed a nodular opacity in the apical right lung (Table 2) and computed axial tomography highlighted a nodular apical ridge of the upper left lobe, compatible with a specific granuloma and a diffuse pleural thickening. The patient scored positive with the Quantiferon TB GOLD test (IFN-γ values of 1.3 IU ml^−1^ and 5.19 IU ml^−1^ for *M. tuberculosis-*specific antigens and mitogen, respectively). The patient was then placed on suspicion of TB infection and transferred to the Infectious Diseases Unit and hospitalized in respiratory isolation. As indicated in Table 2, microbiological examination (AFB staining, culture and genome detection) for mycobacteria was conducted on sputum, stool, urine and biopsy material. AFB smears and genome detection (using Anyplex plus MTB/NTM detection, Seegene) were negative for all previously indicated clinical specimens. Cultural analysis results were negative in all samples with the only exception being the biopsy tissue from the right hand fourth finger that resulted in a positive culture of *M. tuberculosis*, which was later found to be susceptible to all the first line anti-mycobacterial drugs. This results was evidence of cutaneous TB disease and the patient was started on first-line anti-TB medications. A slow clinical improvement of the affected finger was obtained throughout the 9 months of medical treatment, which however did not rule out a surgical intervention.

**Table 2.** Schematic information on clinical health state of patients versus mycobacteria research QFB, quantiferon Gold TB test; Rx, X-Rays.

	Age/Sex	Immunological response		Rx	Mycobacterial search		
		TST	QFB		AFB	PCR	Positive culture
Patient 1	26/F	nd	+	+	−	−	From biopsy material
Patient 2	77/M	nd	−	−	−	−	From biopsy material

### Patient 2

A 76-year-old Italian man, with a clinical history of a highly aggressive prostate cancer treated with hormonal chemotherapy (bicalutamide plus triptorelin), was referred due to the onset about 12 months before of a nodular sore at the right wrist which was previously treated with steroids and physiatric therapy without any clinical improvement and subsequently, following lesion enlargement and fistulation with discharge of purulent material on which a bacterial culture yielded growth of *Staphylococcus epidermidis*. Targeted antibiotic therapy was unsuccessful and the patient was admitted at the Orthopedic and Hand Surgery Unit of our hospital and underwent surgical dorsal synovectomy of the right wrist (both joints and tendons). The histological examination revealed a necrotic giant cell granulomatous lesion. AFB and detection of mycobacterial genomic DNA (using Anyplex plus MTB/NTM detection, Seegene) performed on biopsy tissue gave negative results, as did the cultural exam for mycobacteria. No symptoms or signs of pulmonary involvement were observed and a chest X-ray was negative for pulmonary infiltrates. Due to the results of histological analysis of biopsy tissue, the patient was transferred to the Infectious Diseases Unit and placed in respiratory isolation. Microbiological examination for the detection of mycobacteria was carried out on sputum, stools, urine and bronchoalveolar lavage, all providing negative results (Table 2). The Quantiferon TB GOLD test was negative (0.010 IU ml^−1^ of IFNγ to *M. tuberculosis* antigens), with a weak response to mitogen stimulation (0.59 IU ml^−1^ IFNγ). A positive culture for *M. tuberculosis* was subsequently obtained from the biopsy specimen of the skin lesion, confirming cutaneous TB. The patient was started on four first-line anti-TB medications (rifampin, isoniazid, pyrazinamide and ethambutol), and improvement of the skin lesion was observed.

## Discussion

Cutaneous TB is an uncommon disease caused by *M. tuberculosis* that can be difficult to diagnose because of the non-specific clinical features of the lesions. Different factors contribute to this disease, in particular the increasing presence of immunocompromised subjects due to HIV infection or cancer treatment (Bravo & Gotuzzo, 2007). The two cases presented, highlight the complexity associated with the pathogenesis and diagnosis of cutaneous TB.

Patient 1 showed a skin lesion, following a domestic incident that had occurred two-months earlier and 4 months after giving birth. Anamnesis led to suspicion of mycobacterial infection that was later confirmed by microbiological analysis, though contact with a patient with active pulmonary TB was ruled out. It is well known that near-pregnancy condition is a risk factor for mycobacterial infection and specifically for TB reactivation (Böddinghaus *et al.*, 2007; Good *et al.*, 1981) because of a weakening of the host immune status. As mentioned before, latent TB is characterized by a dynamic equilibrium between the host immune system and *M. tuberculosis* that can persist in many tissues throughout the body (Delogu & Goletti, 2014). In this context, the local inflammation caused by the herringbone may have conveyed pre-infected macrophages or other cells to the injury site that, given the transient immune deficit, may have provided the proper environment for *M. tuberculosis* replication (Goletti *et al.*, 2014; Lowe *et al.*, 2012).

Patient 2 showed an aggressive synovitis of the right wrist which did not improve after antibacterial treatment. Despite the clinical examinations not revealing the classical pulmonary TB signs and the immunological test turning out to be negative, a strong suspicion of *M. tuberculosis* involvement, subsequently confirmed by microbiological culture, was formed because of the histopathological evidence of a granulomatous lesion in the skin tissue involved. The patient was suffering from cancer, which represents a risk factor for cutaneous mycobacterial infections (Handog *et al.*, 2008; Santos *et al.*, 2014). Moreover, the severe immunosuppression status, often generated following chemotherapic treatments, may have been responsible for the negative result obtained with the Quantiferon, though IGRAs cannot be used to rule out active TB (Goletti *et al.*, 2014). Despite the great difficulty in distinguishing endogenous from exogenous TB, the anamnestic and clinical features are idicative of an endogenous cutaneous TB manifestation for patient 1, while it is not possible to determine the pathogenetic mechanism leading to cutaneous TB for patient 2.

The paucibacillary nature of the lesions and the difficulties in extracting mycobacteria from biopsy material, as highlighted by the latter case described, make the microbiological confirmation of cutaneous TB challenging. However, in both cases the combined use of microbiological tests, accurate anamnesis and clinical observations have resulted in first a suspicion and then a final diagnosis of cutaneous TB. Rapid detection of *M. tuberculosis* may be a key step for the diagnosis of some forms of cutaneous TB, where the manifestations could progress to long-term serious complications, which may include the development of squamous cell carcinoma, or lead to surgical amputation of the affected area, as for our patient 1.
